# Lupus systémique et atteinte rénale: apport des anticorps anti-SSA

**DOI:** 10.11604/pamj.2015.20.39.5505

**Published:** 2015-01-14

**Authors:** Kenza Baline, Karim Zaher, Hassan Fellah, Hakima Benchikhi

**Affiliations:** 1 Service de Dermatologie et de Vénéréologie, CHU Ibn Rushd, Casablanca, Maroc; 2 Laboratoire d'Immunologie, Faculté de Médecine et de Pharmacie, Casablanca, Maroc

**Keywords:** Lupus systémique, néphropathie lupique, auto-anticorps, anticorps anti-SSA, systemic lupus, lupus nephritis, autoantibody, anti-SSA

## Abstract

Le but de notre travail est de déterminer le profil des auto-anticorps chez 30 patients ayant un lupus systémique avec ou sans atteinte rénale afin d’établir une corrélation clinico-immunologique entre la néphropathie lupique et ces auto-anticorps. Il s'agit d'une étude transversale de 30 patients atteints de lupus érythémateux systémique diagnostiqués au service de dermatologie durant la période de Décembre 2010 à Décembre 2012 et réalisée conjointement avec le laboratoire d'immunologie. Les anticorps anti-ADN étaient retrouvés chez 17 patients (56.7%) suivis des anti-SSA dans 12 cas (40%). Cinq patients (62.5%) ayant une atteinte rénale avaient des anticorps anti DNA négatifs. Parmi ces patients avec atteinte rénale, 37.5% avaient des anticorps anti SSA sans anticorps anti DNA. La moitié des patients ayant une atteinte rénale (50%) avaient des anticorps anti SSA positifs. Notre série montre l'importance des anticorps anti-SSA surtout chez des patients avec des anticorps anti-DNA négatifs non seulement pour le diagnostic du lupus systémique mais aussi pour déceler certaines manifestations systémiques comme l'atteinte rénale.

## Introduction

Le lupus érythémateux systémique (LES) est une maladie auto-immune caractérisée par la production d'un large panel d'auto anticorps: les anticorps antinucléaires particulièrement les anticorps anti-DNA natifs. De nombreuses publications ont établi une corrélation clinico-immunologique de patients atteints de LES, mais peu d’études se sont intéressées de manière précise à la prévalence des différents auto-anticorps en cas d'atteinte rénale. En effet, la néphropathie lupique est fréquente dans l’évolution du lupus systémique (20 à 50% des cas selon les séries) et influence considérablement le pronostic fonctionnel et vital de cette pathologie [1]. La survie à 20 ans chute de 81% en l'absence de néphropathie lupique à 61% en sa présence [2]. Il conviendrait donc de se doter de moyens pertinents pour suivre les malades en quête d'une éventuelle atteinte rénale. Le but de notre travail est donc de déterminer le profil des auto-anticorps chez 30 patients ayant un lupus systémique avec ou sans atteinte rénale et de préciser l'apport de ces auto-anticorps dans le diagnostic de la néphropathie lupique.

## Méthodes

Il s'agit d'une étude transversale de 30 patients atteints de lupus érythémateux systémique diagnostiqués au service de Dermatologie au CHU Ibn Rochd de Casablanca. Elle a été réalisée conjointement avec le laboratoire d'immunologie et a été menée sur une période de deux années (Décembre 2010-Décembre 2012). Les patients remplissaient au minimum quatre critères de l'American Collège of Rheumatology (ACR) [3]. Une fiche d'exploitation a été établie pour chaque patient et concernait les antécédents, les données cliniques (rash malaire, photosensibilité, phénomène de Raynaud, atteinte rénale, atteinte rhumatologique, atteinte neurologique…) et les données biologiques (troubles hématologiques, vitesse de sédimentation, protéinurie de 24h, AAN, AC anti DNA, anti SSA, anti SSB, anti SM, anti RNP, nucléosome, histone). Nous avons réalisé un prélèvement sanguin pour tous les patients après leur consentement. L’échantillonnage était systématique pour tous les patients qui ont consulté durant cette période et qui remplissaient les critères d'inclusion. Les anticorps antinucléaires (AAN) ont été recherchés par la technique d'immunofluorescence indirecte. Les anticorps anti DNA ont été dosés par la technique ELISA et les autres auto-anticorps par une technique immunoenzymatique de type immunodot. Toutes ces analyses étaient réalisées au laboratoire d'immunologie de la faculté de médecine et de pharmacie de casablanca. Les dosages des auto-anticorps ont été effectués à but diagnostique et thérapeutique. Nous avons déterminé le profil des auto-anticorps sus-cités chez les patients lupiques avec ou sans atteinte rénale et l'analyse statistique a été réalisée par Epi info 3.5.1.

## Résultats

**Figure 1:**
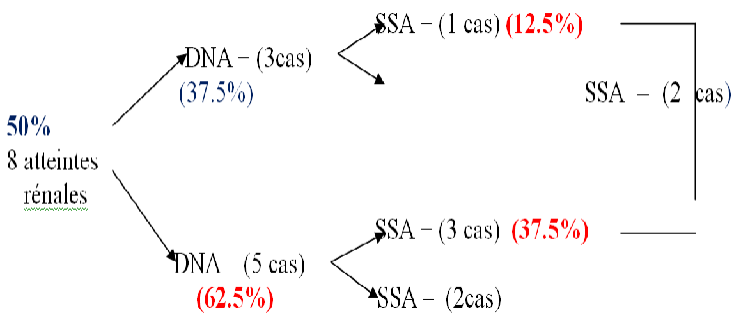
Pourcentages des anti-DNA et anti-SSA chez les Patients lupiques avec atteinte rénale

Parmi les 30 patients, 26 étaient de sexe féminin et 4 de sexe masculin avec un sexe ratio H/F de 0.15. La moyenne d’âge était de 35 ans avec des extrêmes de 14 à 59 ans. Les manifestations cutanées étaient observées chez 29 malades (96.7%) et l'atteinte hématologique chez 28 patients (93,3%). Huit patients avaient une atteinte rénale soit 26.7%. La néphropathie lupique a été classée de type III de l'OMS dans 2 cas, de type IV dans 4 cas et de type V dans 2 cas. Les AAN ont été positifs chez 25 des 30 patients, avec un aspect moucheté (40%) et homogène (23.3%). Les anticorps anti-ADN étaient retrouvés chez 17 patients (56.7%) suivis des anti-SSA dans 12 cas (40%), anti SM/RNP dans 7 cas (23.3%), anti SSB et anti nucléosome chez respectivement 6 cas (20%), anticorps antihistone dans 3 cas (10%) et anticorps anti SM dans 2 cas (6.7%). Cinq patients (62.5%) ayant une atteinte rénale avaient des anticorps anti DNA négatifs (Tableau 1). Parmi ces patients avec atteinte rénale, 37.5% avaient des anticorps anti SSA sans anticorps anti DNA (Figure 1). La moitié des patients ayant une atteinte rénale (50%) avaient des anticorps anti SSA positifs (Tableau 2).

**Tableau 1:** Prévalence des anti-DNA dans la néphropathie lupique

	Atteinte rénale	Pas d'atteinte rénale
Ac anti DNA +	3	14
Ac anti DNA -	5	8

**Tableau 2:** Prévalence des anti-SSA dans la néphropathie lupique

	Atteinte rénale	Pas d'atteinte rénale
SSA +	4	8
SSA -	4	14

## Discussion

Dans cette série, la moitié des patients atteints de lupus systémique n'avaient pas d'anticorps anti-DNA. Dans ce groupe, parmi ceux ayant une atteinte rénale, 37.5% avaient des anticorps anti-SSA positifs. Ainsi, l'apport des anticorps anti-SSA parait fondamental en cas de lupus systémique avec atteinte rénale. Certes, notre série se heurte au biais du faible échantillonnage, néanmoins; il s'agit d'une étude prospective menée dans un service de dermatologie ayant utilisé un panel d'auto-anticorps représentatif. Ceci a permis par conséquent une comparaison plus large et une corrélation clinico-immunologique entre différents auto-anticorps en matière de néphropathie lupique. Dans notre série, l'atteinte rénale était notée dans 26.7% des cas qui étaient dans leur majorité des stades avancés IV et V (75%). La fréquence de cette atteinte rénale dans notre contexte est en accord avec les données de la littérature (20 à 50% des cas selon les séries) [1]. Cette atteinte conditionne le pronostic fonctionnel mais aussi vital de la maladie. Il s'agit d'un facteur pronostique statistiquement significatif de mortalité dans une série marocaine de 129 patients, ce qui est d'ailleurs en accord avec les données de la littérature [4]. Ceci a justifié une étude détaillée du profil des auto-anticorps chez les patients lupiques ayant une atteinte rénale dans notre série. Diverses études ont signalé l'intérêt primordial des anti-DNA pour le diagnostic et le suivi de la néphropathie lupique. Un excès d'anticorps anti DNA précède une exacerbation et la persistance de taux élevés signe une poussée de néphropathie lupique [5, 6]. Les Anticorps (Ac) anti DNA ressortent dans 56.7% dans notre série (35 à 98% dans la littérature). Cependant, ils étaient absents chez 62.5% des patients ayant une atteinte rénale au profit des anti SSA qui étaient positifs chez la moitié des patients ayant une atteinte rénale (50%) et présents même en l'absence d'anti-DNA dans 37.5% des cas. En effet, certains auteurs ont déjà montré que les autres Ac anti-nucléaires peuvent apporter en l'absence des Ac anti DNA un élément diagnostique et de surveillance comme les Ac anti SSA et anti Sm [7]. Les Ac anti SSA ont une grande valeur prédictive pour le diagnostic du lupus systémique surtout pour les patients positifs en AAN mais sans anti-DNA ou anti-Sm [7]. Peene et al en analysant le diagnostic clinique de 181 malades ayant dans leur sérum des anti-SSA et/ou anti-SSB ont trouvé que 80% des malades ayant uniquement des anti-SSA s'avèrent être des lupus systémiques [8]. Les atteintes cutanées et la photosensibilité sont les manifestations cliniques les plus fréquentes chez les malades ayant des anticorps anti-SSA. Peu d’études ont documenté la relation anticorps anti-SSA et néphropathie lupique. Simmons-O'Brien et al ont trouvé que 47% des patients ayant une atteinte rénale ont des anticorps anti SSA mais n'ont pas d'anticorps anti-DNA [9]. Ces auteurs ont conclu que les patients avec des Ac anti SSA positifs doivent être régulièrement suivis afin de détecter la survenue de manifestations systémiques. Dans une série tunisienne de 128 patients ayant un lupus systémique, les anticorps anti SSA ont été trouvés chez 7% des patients ayant un lupus systémique grave avec atteinte rénale en l'absence d'anticorps anti DNA natif et anti Sm [10]. Le pourcentage des patients remplissant cette condition dans notre série est de 37.5%. La fréquence des Ac anti SSA varie de 26 à 60% dans la littérature [11] et est de 40% dans notre série. A travers notre série, nous soulignons l'importance déjà mentionnée par des études précédentes des anticorps anti SSA surtout chez des patients avec des Ac anti DNA négatifs non seulement pour le diagnostic du lupus mais aussi pour déceler certaines manifestations systémiques comme l'atteinte rénale.

## Conclusion

Un suivi régulier des patients lupiques avec anticorps anti SSA positifs surtout ceux avec des anti-DNA négatifs s'impose afin de déceler une atteinte systémique notamment rénale d'autant plus que cette manifestation est sévère dans notre contexte.

## References

[1] Karras A (2012). Atteinte rénale du lupus érythémateux disséminé. Presse Med..

[2] Cervera R, Khamashta MA, Font J, Sebastiani GD, Gil A, Lavilla P, Mejía JC, Aydintug AO (2003). Morbidity and mortality in systemic lupus erythematosus during a 10-year period: a comparison of early and late manifestations in a cohort of 1,000 patients. Medicine (Baltimore).

[3] Hochberg MG (1997). Updating the American College of Rheumatology revised criteria for the classification of systemic lupus erythematosus. Arthritis Rheum..

[4] Bouras M, Hali F, Khadir K, Benchikhi H (2014). Lupus érythémateux systémique: mortalité et facteurs de mauvaispronostic dans une série marocaine de 129 cas. Ann Dermatol Venereol..

[5] Ségalen I, Renaudineau Y, Hillion S, Hanrotel C, Le Meur Y, Youinou P (2011). Quels auto-anticorps pour le diagnostic et le suivi de la néphropathie lupique?. Immuno-analyse et biologie spécialisée..

[6] Renaudineau Y, Renaudineau E, Le Meur Y, Chauveau A, Youinou P (2008). Intérêt des nouveaux examens sérologiques pour la néphropathie lupique. Immuno-analyse et biologie spécialisée..

[7] Shrivastava A, Khanna D (2011). Autoantibodies in systemic lupus erythematosus: Revisited. Indian Journal of Rheumatology..

[8] Peene I, Meheus L, Veys EM, De Keyser F (2002). Diagnostic association in large and consecutively identified population positive for anti-SSA and/or anti-SSB: the range of associated diseases differs according to the detailed serotype. Ann Rheum Dis..

[9] Simmons-O'Brien E, Chen S, Watson R, Antoni C, Petri M, Hochberg M, Stevens MB, Provost TT (1995). One hundred anti-Ro (SS-A) antibody positive patients: a 10 year follow-up. Medicine (Baltimore).

[10] Ghedira I, Sakly W, Jeddi M (2002). Caractéristiques cliniques et sérologiques du lupus érythémateux systémique: à propos de 128 cas. Pathol Biol(Paris).

[11] Haddouk S, Ben Ayed M, Baklouti S, Hachicha J, Bahloul Z, Masmoudi H (2005). Autoanticorps dans le lupus érythémateux systémique: profil et corrélations cliniques. Pathol Biol(Paris).

